# Genetic characterization and evolution of H6N6 subtype avian influenza viruses

**DOI:** 10.3389/fmicb.2022.963218

**Published:** 2022-08-01

**Authors:** Mingxian Cui, Yanming Huang, Xingbo Wang, Xiyi Bian, Liuyang Du, Yan Yan, Jinyan Gu, Weiren Dong, Jiyong Zhou, Min Liao

**Affiliations:** ^1^MOA Key Laboratory of Animal Virology, Department of Veterinary Medicine and Center of Veterinary Medical Sciences, Zhejiang University, Hangzhou, China; ^2^Collaborative Innovation Center and State Key Laboratory for Diagnosis and Treatment of Infectious Diseases, The First Affiliated Hospital, Zhejiang University, Hangzhou, China

**Keywords:** H6N6-subtype AIVs, genetic evolutionary, reassortment, SA receptors, glycosylation sites

## Abstract

H6-subtype avian influenza virus (AIV) was prevalent in the world and could sporadically infect humans. Here, a new chicken-derived H6N6-subtype AIV strain A/chicken/Zhejiang/49/2021 (ZJ49) was isolated in Zhejiang Province, China in 2021. Phylogenetic analysis by Maximum likelihood methods showed that H6-subtype AIVs were classed into 13 groups according to HA gene. The ZJ49 strain belonged to the G12 group, which mainly consisted of strains from Asian and dominated in recent years. Based on NA gene, H6-subtype AIVs were divided into N6.1 and N6.2 clades according to the NA gene. The ZJ49 isolate was located in the N6.2e clade, which mainly consisted of the H5N6-subtype AIVs. Phylogenetic analysis by Bayesian methods showed that the effective quantity size of H6-subtype AIVs increased around 1990, reached a peak around 2015, declined after 2015, then kept in a stable level after 2018. The reassortment analysis predicted that the PB2, PA, and NA genes of ZJ49 may recombine with H5-subtype AIVs. The amino acid at 222 position of HA gene of ZJ49 strain mutated from A to V, suggesting that ZJ49 has a potential ability to cross species barriers. The four glycosylation sites were highly conserved, implying less impact on the fold and conception of HA stem structure. Our results revealed the complicated evolution, reassortment, and mutations of receptor binding sites of H6-subtype AIVs, which emphasize the importance to continuously monitor the epidemiology and evolution of H6-subtype AIVs.

## Introduction

Avian influenza is a kind of avian infectious disease caused by the avian influenza viruses (Avian Influenza viruses, AIVs) characterized by respiratory system lesions or systemic sepsis. AIVs can be divided into low pathogenic AIVs (LPAIVs) and high pathogenic AIVs (HPAIVs; Germeraad et al., 2019). Compared with HPAIVs, LPAIVs caused continuous effects to chickens in reducing egg production and even became a potential threat to human public health (Wang et al., 2014). H6-subtype AIVs belong to LPAIVs (Lin et al., 2020; Li et al., 2021). The H6 subtype AIV was first isolated from a turkey in 1965 (Munster et al., 2007; Rimondi et al., 2011), and subsequently from shorebirds and wild ducks (Downie et al., 1973; Sharp et al., 1993). Currently, the H6 viruses became one of the most predominant virus subtypes circulating in wild birds and domestic poultry throughout different continents (Hoque et al., 2015; Lee et al., 2017; Rimondi et al., 2018) and they were undergoing constant reassortment with different subtypes of viruses (Yuan et al., 2016; Kumar et al., 2018; Li et al., 2019). H6-subtype AIVs highly occurred in China as compared to other countries. Five subtypes of H6 influenza virus (H6N1, H6N2, H6N5, H6N6 and H6N8) co-circulated in Eastern China (Zhao et al., 2011), which formed a significant part of the natural influenza virus reservoir in domestic ducks. In China, the most representative strain, A/teal/Hong Kong/W312/97 (W312), was isolated during the “bird flu” incident in Hong Kong in 1997 (Cheung et al., 2007). The duck-derived H6N2 viruses were most frequently detected in China from 2002 to 2008, then the H6N6 viruses became the most epidemic subtype since 2009 (Zhao et al., 2011). H6N6 viruses had become the main circulating H6 subtype AIVs from 2014 to 2018 (Li et al., 2019; Cui et al., 2021).

Remarkably, H6 subtype AIVs can not only infect birds but also infect mammals. A strain of H6N6 was isolated from swine in Yangzhou, Eastern China in 2009 (Zhao et al., 2013). The first human infection with the A/Taiwan/2/2013 (H6N1) was reported on June 21, 2013 (Shi et al., 2013; Wei et al., 2013). Due to the lack of a commercial available vaccine, H6-subtype AIVs had continuously evolved and diversified into many distinct lineages in China and other Asian countries (Huang et al., 2010, 2012). These findings suggest that H6 subtype AIVs could cross the species barrier and infect mammals, including humans. Antigenic variation in the influenza virus had the potential to cause rapid adaptation and led to an unexpected evolutionary road (Liu et al., 2013; Morens et al., 2013). Therefore, extensive monitoring of influenza virus variation is necessary for understanding of the epidemilogy of influenza virus. Here, a new H6N6-subtype AIV strain from chickens named A/chicken/Zhejiang/49/2021 (ZJ49) was isolated in Zhejiang, China in 2021. The genetic evolutionary, reassortment, and mutations of receptor binding sites of H6-subtype AIVs were analyzed to investigate the genetic evolution regulations and molecular characteristics. Our findings revealed that H6N6-subtype AIVs were likely to spread across species and recombine with highly pathogenic viruses.

## Materials and methods

### Sample collection and virus isolation

To investigate the prevalence of AIVs in Zhejiang Province, fixed location surveillance in poultry farms were conducted in four cities of Zhejiang Province in 2021. A total of 923 oropharyngeal and cloacal swabs including 810 from chickens and 113 from ducks were collected from apparently healthy poultry from four cities in Zhejiang, including Jiaxing (*n* = 389), Huzhou (*n* = 294), Zhoushan (*n* = 100), and Taizhou (*n* = 140). The samples in Zhoushan were free-range samples from farmers. The total RNA extracted from the samples was detected by RT-PCR using universal primers targeting the M gene (M-F: CGTAGACGCTTTGTCCAAAATGCC; M-R: AAGACGATCAAGAATCCACAATA). All RT-PCR positive samples were isolated with 10-day-73 old SPF chicken embryos for 96 h at 37 °C for virus isolation. Hemagglutinin-positive samples were further confirmed by RT-PCR with above primers M-F and M-R. The isolate was serially passaged on chicken embryos and subjected to extract RNA for Next-generation sequencing (NGS).

### Whole-genome sequencing of AIV isolates and sequencing data assembly

The ultra-concentrated virus was sent to Genergy Bio-Technology (SHANGHAI) CO., LTD for Next-generation sequencing. The main methods were as follows: NGS (van Dijk et al., 2014) was used to determine the whole-genome sequences of AIV isolates. For NGS, the viral RNAs were quantified using the 2,100 Bio-analyzer System (Agilent Technologies). RT-PCR and cDNA synthesis were conducted using the Prime Script One-Step RT-PCR kit (Takara), with influenza A random primer. The sequencing libraries with an insert size of 200–400 bp were prepared by end-repairing, dA-tailing, adaptor ligation, and PCR amplification, all according to the instructions provided by the manufacturer (Illumina). The libraries were sequenced on an Illumina NovaSeq 6,000 Sequencer (Dillies et al., 2013) by 150 bp paired-end sequencing, and sequencing depth for AIVs isolates was 2G per sample.

NGS sequencing reads were processed and assembled as described previously (Yu et al., 2014). The filtered reads were mapped to the Influenza database to choose the best-matching reference sequences and determine genotypes. Burrows-Wheeler Aligner (BWA version 0.7.17; Li and Durbin, 2009) and SAMtools (version 1.10; Li et al., 2009) were then used to perform the reference-based assembly.

### Sequence retrieval and phylogenetic reconstruction

In order to describe the spatiotemporal evolution of H6-subtype AIVs, the H6NX and HXN6 influenza viruses were focused in this study. The data of the global distribution of H6NX are listed in Supplementary Table S1. The gene sequences of isolates with the full-length gene sequences of all H6NX HA gene sequences, HXN6 NA gene sequences, and all H6N6 internal fragments in the GISAID and Genbank Influenza Research Databases were combined. Identical sequences were removed through RAxML-ng. Sequences derived from laboratory strains and containing ambiguous characters were also removed from the dataset. The Visual Studio code was used to remove the noncoding region and to keep the full length of the ORFs encoding each fragment genes. Only the longest ORFs were retained for each sequence. Nucleotide sequences were aligned using MAFFT (Katoh et al., 2002). Identical sequences were removed again by RAxML-ng. The total number of sequences for HA, NA, MP, NP, NS, PA, PB1, and PB2 were 2063, 3,338, 386, 386, 376, 308, 259, and 266, respectively. The accession number and the sequence source were listed in Supplementary Table S2.

All HA sequences from GISAID and Genbank were used to determine the distribution ratios of various subtypes across continents. Coding region sequences (CDS) were used for phylogenetic reconstruction using RAxML v8.2.4 (Stamatakis, 2014) based on the general time-reversible model and gamma distribution (GTR + G) model. The auto MRE bootstrapping convergence criterion (Pattengale et al., 2010) was applied to determine the most suitable number of replicates instead of the default 1,000 replicates. The selection of most suitable replicates of bootstrap was done as follow: after 50 replicates, all of the generated bootstrapped trees were repeatedly (1,000 permutations) split into two equal subsets and the Weighted Robinson–Foulds (WRF) distance was calculated between the majority-rule consensus trees of both subsets (for each permutation). Bootstrapping convergence was considered to be reached if over 99% of permutations have low WRF distances (<3%). In this case, convergence was reached after 400, 650, 900, 1,000, 750, 550, 420, and 550 replicates.

The phylogenetic tree was visualized using iTOL v4 (Letunic and Bork, 2016). For further analysis, the big HA tree was split into several different groups. The criteria for group selection were as follows: Collapse all clades whose average branch length distance to their leaves is below 0.15. The naming of branches and sub-branches in the NA evolutionary tree follows the topological rules of the evolutionary tree: The naming of primary branches follows “name of subtype/gene + ‘.’ + number,” Subclades are named with alternating letters and numbers, such as H5.2a. Try to follow the order of host (avian > human > swine > horse > dog) > space (Western hemisphere > Eastern hemisphere) > time (early period > later period).

### Evolutionary and reassortment analysis

The TempEst was used to assessed temporal signal in our data sets (Rambaut et al., 2016) and Path-sampling and Stepping-stone estimation approaches were used to assess the best fitting model through marginal likelihood estimation (Baele et al., 2013, 2016). The BEAST package (v2.6.6) was used to construct the maximum clade credibility (MCC) tree (Suchard et al., 2018). The best-fit nucleotide substitution model was selected using IQ-tree (Nguyen et al., 2015). We selected GTR + G distributed rate variation among sites in nucleotide substitution model, uncorrelated relaxed lognormal clock set in the molecular clock, Coalescent: Bayesian Skyline set in prior, total chain length was 4 × 10^8^ and sampling every 1,000 steps. Four dependently runs were combined using Logcombiner. In addition, we used a coalescent-based nonparametric skyline prior to the tree topologies to model the effective avian size over time.

The reassortment events among the H6N6-subtype and other subtype viruses were carried out with the sequences which information were listed in Supplementary Table S3. ML phylogenetic trees inferred for HXN6-subtype AIVs HA and NA using RAxML under the general time-reversible substitution model with gamma-distributed rates across sites. In total, 1,000 bootstraps were evaluated to assess support values. Full genomes sequences were analyzed separately to detect reassortment events using RDP5 (He et al., 2020) and Similarity plot (Soli et al., 2019). A total of eight methods including RDP, GENECONV, 3Seq, Chimaera, SiScan, MaxChi, BootScan, and LARD implemented in RDP5 were applied. Reassortment detected by at least three of the eight methods with a value of *p* cut off of 0.05 was considered as true reassortment (Sabir et al., 2016).

### The haemagglutinin binding site and N-glycosylation site

Amino acid glycosylation sites of ZJ49 isolate were predicted by NetNGlyc-1.0 prediction server^1^ (Wang et al., 2006). The tertiary structure model (The template was 4wss.1.A) of ZJ49 HA protein was constructed through the SWISS-MODEL online server^2^ (Waterhouse et al., 2018), and PyMOL (Version 2.0.7) was used for visual analysis to map the spatial location of receptor binding sites and glycosylation sites (Lu, 2020).

## Results

### Spatial distribution of the H6-subtype influenza virus

**Figure 1:**
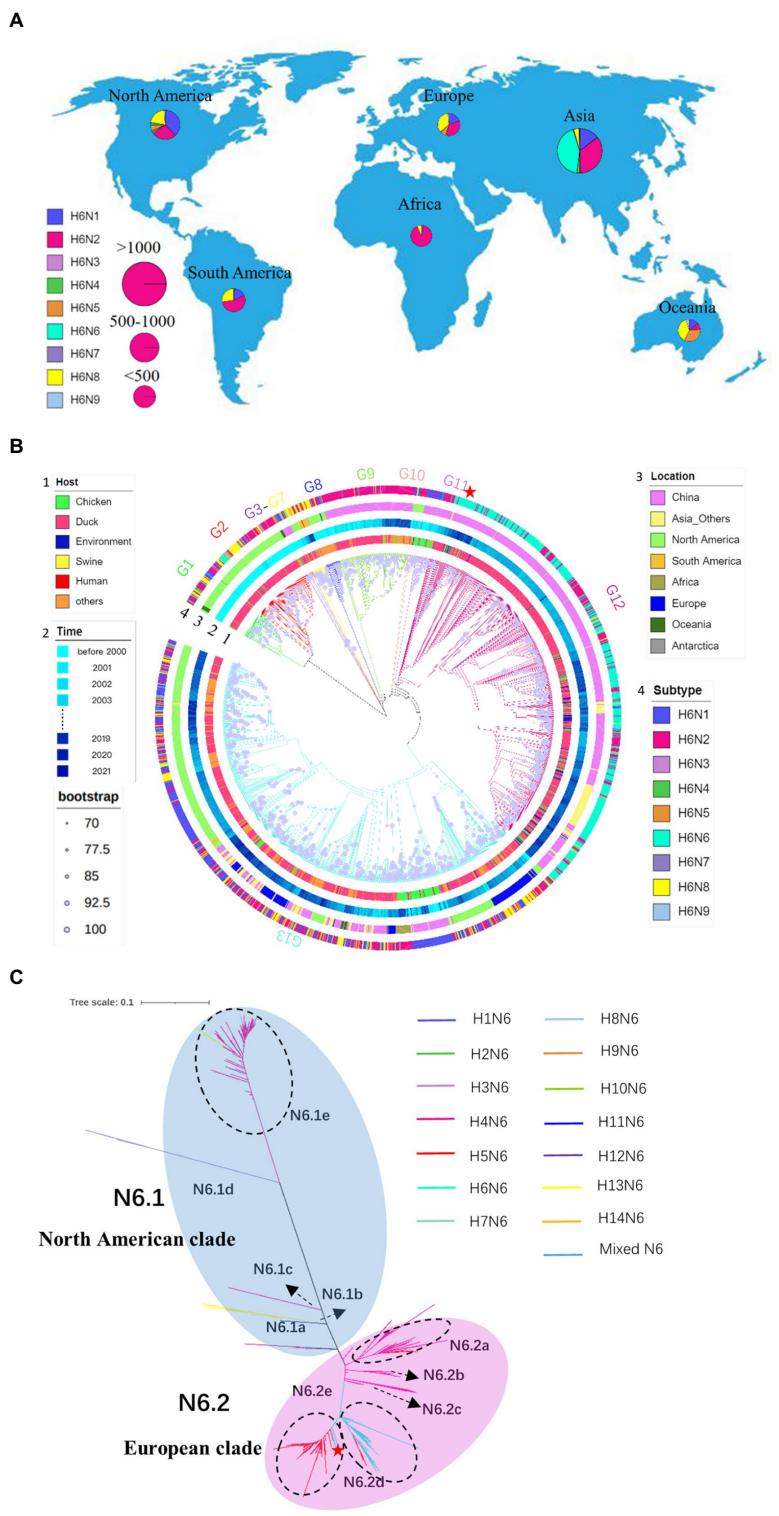
Spatial distribution of the H6-subtype influenza virus. **(A)** Global distribution of H6NX isolates. The proportion of isolates for each subtype category is visualized in a pie chart. The size of the pie chart is proportional to the number of isolates in each continent. **(B)** Phylogeny and groups of H6NX HA. Only bootstrap values≥70% are visualized as a purple circle in the middle of the branch. The size of the circle is proportional to the bootstrap value. Categories of virus (such as subtype, host, location, time) are identified by different colors of circles in the outer part of the tree. Phylogenetic independent groups are indicated with different colors of branches. **(C)** Phylogeny and groups of HXN6 NA. Tree scale is 0.1. Different subtypes are represented by different colored branches. ZJ49 is indicated by a red five-pointed star.

In 2021, a total of 923 pharyngeal and anal swabs were collected from Zhejiang Province, China. A total of 71 positive samples were detected, with a positive rate of 7.69% for AIV detection. Among them, the AIV positive rates of samples from Jiaxing, Huzhou, Zhoushan and Taizhou were 4.88% (19/389), 8.16% (24/294), 28% (28/100) and 0 (0/140), respectively. A strain of chicken-derived H6N6 virus (A/chicken/Zhejiang/49/2021, ZJ49) was isolated from the samples of Huzhou. The nucleotide sequence lengths of PB2, PB1, PA, HA, NA, NP, M, and NS fragments of ZJ49 were 2,280, 2,274, 2,151, 1701, 1,380, 1,497, 982, and 838 (Supplementary Table S4). Total of 2064 H6NX genome sequences including ZJ49 were collected from GISAID and Genbank (Supplementary Table S1), which strain informations were subjected to analysis of spatial distribution of H6NX subtype AIVs. There were differences in the proportion of isolates of each H6NX subtype in each continent, with the highest proportion of H6N6 isolates in Asia (about 43.25%; Figure 1A). H6N6-subtype AIVs were found in the three continents including Asia, Europe, and North America, with the highest distribution in Asia (about 96.73%; Figure 1A).

According to the phylogenetic tree (Figure 1B), 13 genetically distinct groups based on HA genes were identified. Most of the strains in the H6NX ML tree were isolated from ducks, indicating that ducks are the main host for H6-subtype AIVs. The number of strains originating from chickens was relatively small, and they were mainly distributed in China. The strains isolated in the early 1970s were mainly concentrated in North America to form G1 and G2 groups. H6N2 and H6N6-subtype AIVs co-evolved and were mainly distributed in the Asia continent especially China. The ZJ49 isolate was located in the G12 group and was most genetically related to A/duck/Hunan/2.06_YYGK86J3-OC/2018 (H6N6), which was consistent with the analysis by BLAST (Figure 1B; Supplementary Table S4). The genome nucleotide homology between these two strains was 98.53% (Supplementary Table S4). The NA subtypes of the G13 strains were more abundant and mainly composed of Eurasian strains and American strains. The strain of H6N1 isolated from human was located in this group.

The NA genes of the N6-subtype AIVs can be divided into two primary branches, N6.1 and N6.2, corresponding to the branches dominated by North America and Eurasia, respectively (Figure 1C). The North American clade was mainly composed of the H4N6-subtype AIVs, and the European clade was jointly composed of H4N6, H5N6 and H6N6-subtype AIVs. Five secondary clades were named N6.1a ~ e, within the N6.1 clade. The N6.1a ~ d branches correspond to the HXN6-subtype AIVs isolated earlier. Five secondary clades were named N6.2a ~ e within the N6.2 branch. The N6.2a ~ c branch was mainly composed of H4N6. N6.2d and N6.2e were dominated by H6N6 and H5N6, respectively. Among them, H4N6 and H5N6 were cross-distributed in N6.2a, and H5N6 and H6N6 were cross-distributed in N6.2d and N6.2e, suggesting that H5N6 may recombine with H4N6 and H6N6. The ZJ49 isolate was located in the N6.2e branch, which was mainly consisted of the H5N6-subtype AIVs.

**Figure 2:**
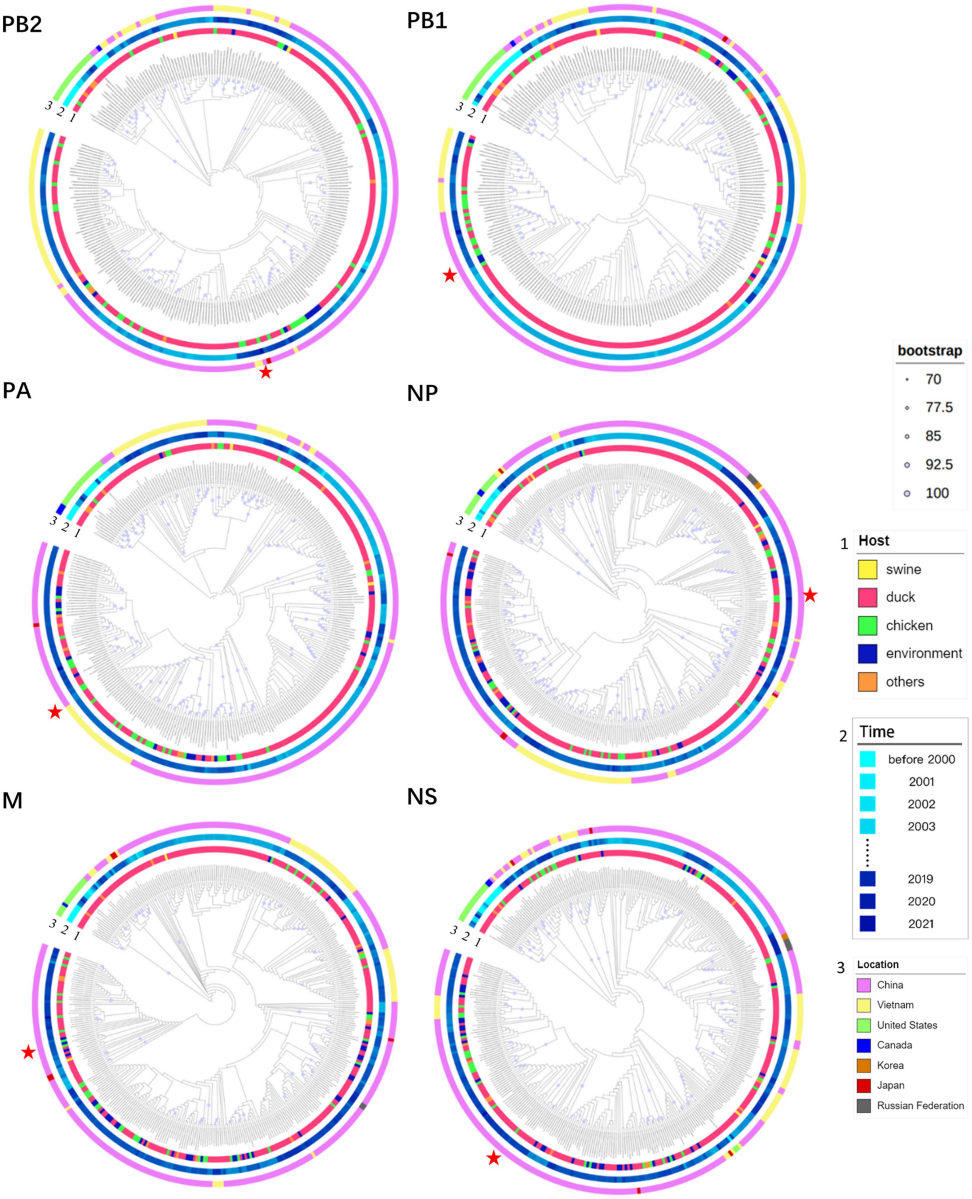
Phylogeny and groups of PB2, PB1, PA, NP, M, and NS of H6N6. Only bootstrap values≥70% are indicated as a purple circle in the middle of the branch. The size of the circle is proportional to the bootstrap value. Categories of viruses (such as host, and location) are identified by different colors. ZJ49 is indicated by a red five-pointed star.

According to the ML trees constructed based on the internal fragments of H6N6 (Figure 2), the distribution of H6N6 can be divided into the seven countries, including China, Viet Nam, United States, Canada, Korea, Russian Federation, and Japan. The results were consistent with the results of phylogenetic analysis based on the HA gene, in which the H6N6-subtype AIVs was mainly prevalent in China.

### Temporal distribution of the H6-subtype influenza virus

**Figure 3:**
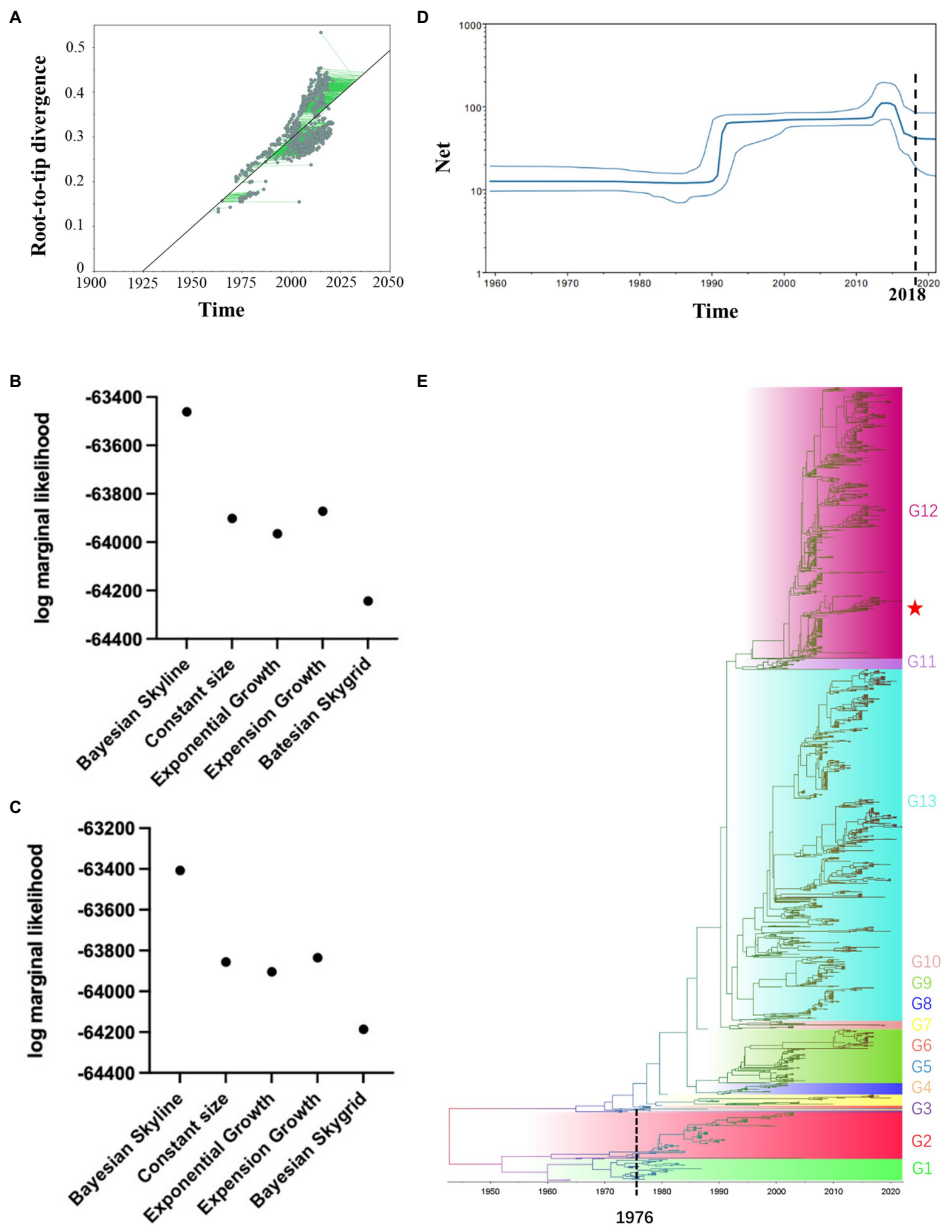
Temporal distribution of the H6-subtype influenza virus. **(A)** Root-to-tip regression analyses. Plots of the root-to-tip genetic distance against sampling time were shown for phylogenies estimated from 2063 HA gene sequences. **(B)** Stepping-stone estimation approaches were used to assess the best fitting model through marginal likelihood estimation. **(C)** Path-sampling estimation approaches were used to assess the best fitting model through marginal likelihood estimation. **(D)** The Bayesian skyline plot was based on the individuals infected by H6NX-subtype AIVs. **(E)** Maximum clade credibility tree of HA gene. The MCC tree was constructed by BEAST (v2.6.6) with GTR + G distribution nucleotide substitution model, relaxed molecular clock, the tree prior was Coalescent: Bayesian Skyline and 4 × 10^8^ total chain length, sample every 1,000 steps.

We explored temporal signal in sequence alignments using TempEst, as assessed by visual inspection and the correlation coefficient, *R*^2^. The result indicated a positive correlation between genetic divergence and sampling time (*R*^2^ = 0.4008) and appeared to be suitable for phylogenetic molecular clock analysis in BEAST (Figure 3A). The Stepping-stone and Path-sampling estimation approaches showed that the log marginal likelihood of Bayesian Skyline was −63461.546 (Figure 3B) and − 63407.094 (Figure 3C), respectively, indicating that the Bayesian skyline was the best fitting model. Due to the increasing number of samples, the effective quantity size increased around 1990, reached a peak around 2015, while declined after 2015, then kept in a stable level after 2018. (Figure 3D). The structure of the MCC tree based on HA was similar to the ML tree. The H6 subtype AIVs were clearly divided into G1–G13 (Figure 3E). Base on the MCC tree, the H6-subtype AIVs spread from North America to Eurasia around 1976. In recent years, H6-subtype AIVs were mainly distributed in G12 and G13 groups. In the NA gene genetic tree, N6.2d mainly consisted of H6N6 strains prevalent in Asia after 2005, and N6.2e mainly consisted of H5N6 strains prevalent in Asia after 2015 (Figure 1C). According to the phylogenetic tree (Figure 2), the ML tree constructed by the internal fragments of H6N6 can be also divided into the early branch and modern branch according to time.

### Reassortment analysis of the H6N6-subtype AIVs

**Figure 4:**
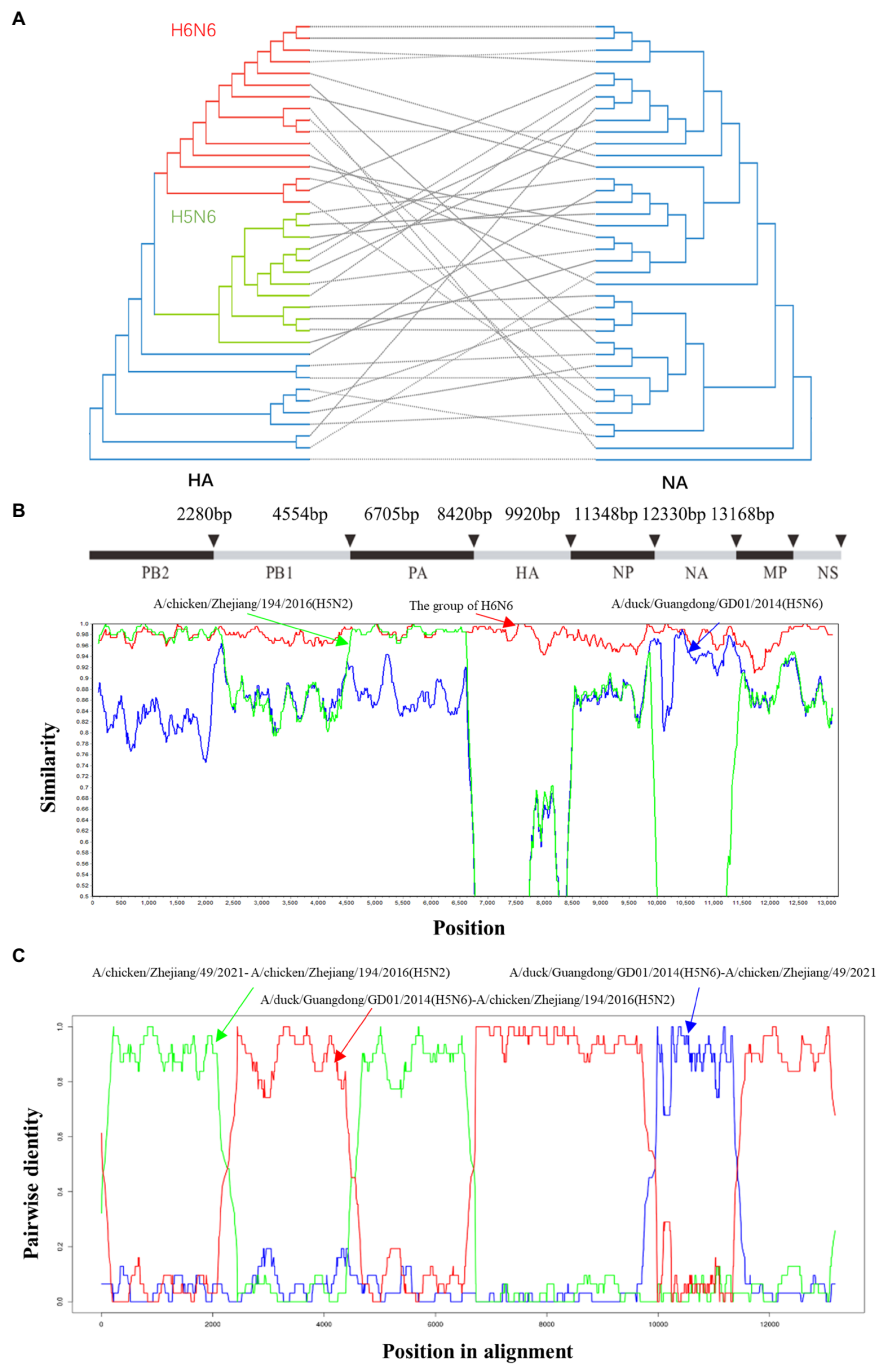
Reassortment analysis on concatenated H6N6 virus genomes. **(A)** ML phylogenetic trees inferred for HXN6-subtype AIVs HA and NA using RAxML under the general time-reversible substitution model with gamma-distributed rates across sites. In total, 1,000 bootstraps were evaluated to assess support values. **(B)** Reassortment analysis was performed using Simplot (v.3.5.1). The H6N6 group mainly included the following strains: A/duck/Jiangxi/10.30_NCNP67B3-OC/2017 (H6N6), A/duck/Ganzhou/GZ151/2016 (H6N6), A/chicken/Hunan/1.12_YYGK22H3-OC/2018 (H6N6), A/environment/Jiangxi/2.06_SRXZJG016-E/2017 (H6N6), A/duck/Hunan/2.06_YYGK86J3-OC/2018 (H6N6), and A/duck/Hunan/2.06_YYGK73J3-OC/2018 (H6N6). **(C)** Reassortment analysis was performed using RDP (v.5.5).

A total of 37 HXN6 genome sequences were collected from GenBank (Supplementary Table S3). The results of reassortment analysis showed that the H6N6-subtype AIVs and H5N6-subtype AIVs recombined frequently (Figure 4A). To explore the reassortment events in ZJ49 isolates, we integrated the sequences that were genetically close to ZJ49 for reassortment prediction. The Simplot results showed that the full genomes sequences of ZJ49 had the highest similarity to the H6N6-subtype AIVs, but the PB2 and PA genes were closer to A/chicken/Zhejiang/194/2016 (H5N2) and the NA gene was closer to A/duck/Guangdong/GD01/2014(H5N6; Figure 4B). Among the RDP detection methods, there were 7 methods (RDP, GENECONV, 3Seq, Chimaera, SiScan, MaxChi, and BootScan) that also can detect the reassortment of ZJ49 (Figure 4C). The results showed that the ZJ49 isolate underwent genetic reassortment with the Major Parent A/duck/Guangdong/GD01/2014(H5N6) and the Minor Parent A/chicken/Zhejiang/194/2016 (H5N2). This result was consistent with the above result showed in Figure 4B. In conclusion, there was a high possibility that the ZJ49 gene recombined with the H5-subtype AIVs.

### Molecular characterization of the H6N6-subtype AIVs

**Figure 5:**
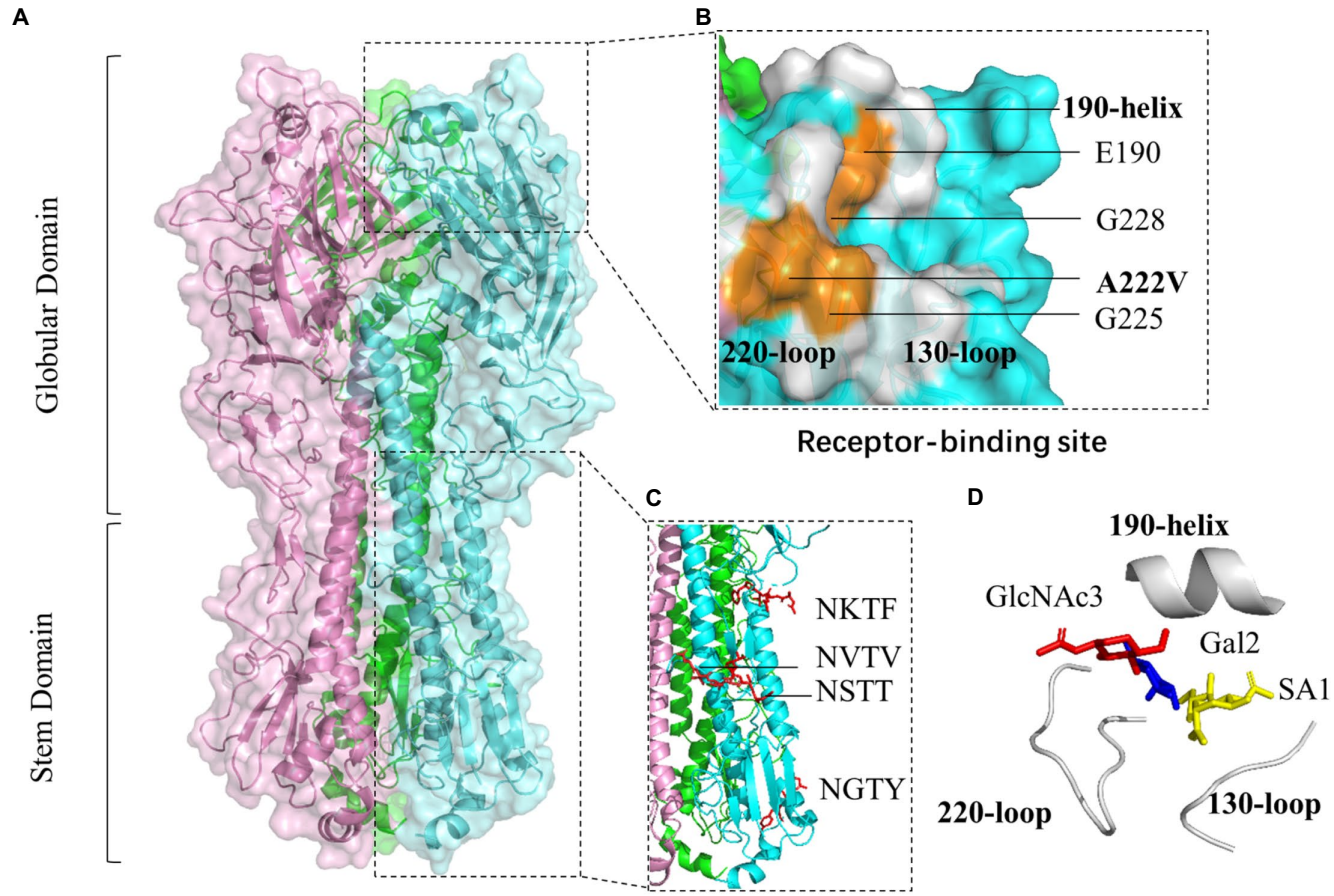
Molecular characterization of the H6N6-subtype AIVs. **(A)** The crystal structure of the ectodomain of haemagglutinin (HA) reveals two distinct domains: the globular domain and the stem domain. Each monomer is represented by pink, green, and blue, respectively. **(B)** Three secondary structural elements (in white color) and the sites associated with H6 receptor-binding properties (in orange color). **(C)** N-glycosylation sites (in red color). **(D)** The H6N6 HA receptor analogue (α2, 3-linked sialylated glycan receptor). Sialylated glycans of the host receptors that influenza viruses bind to contain the three-terminal saccharides: the terminal sialic acid SA1, the galactose ring Gal2 (at position two relative to SA), and N-acetylglucosamine GlcNAc3 (at position three relative to SA).

The length of the ZJ49 HA coding region was 1701 bp, encoding a total of 566 amino acids. The HA trimer projects of ZJ49 can be divided into two domains: the membrane-distal globular domain and the membrane-proximal stem domain (Figure 5A). The results of amino acid variation sites of the HA gene of all H6N6-subtype AIVs were shown in Supplementary Table S5. The result showed that the mutation rate of the G228 site of H6N6-subtype AIVs was 0.95%. Moreover, strains isolated from pigeon, swine, and duck have G228S mutant sites. H6N6-subtype AIVs had a high mutation rate (28.44%) at the A222 site, of which the mutation rate from A to V was 8.40%. The mutation rate of A222V was 15.91% before 2010, 31.82% from 2011 to 2015, and 52.27% after 2015. Moreover, strains isolated from pigeons and swine, and some strains from chickens and ducks have A222V mutant sites. Sequencing by NGS showed that the nucleotide of 222 site of HA gene of ZJ49 mutated from GCT t-o GTT, namely A222V mutation exsists (data not shown). The G228S and A222V played an important role in the cross-species transmission of the H6N6-subtype AIVs. However, there were no corresponding mutations at 190 and 225 sites, which were cross-species related to H6-subtype AIVs. In the newly isolated ZJ49 strain, the E190 site was located on 190-helix, and the V222, G225, G228 sites were all located on 220-loop, except for the mutation from A to V at site 222, the other sites associated with H6 receptor-binding properties were not mutated (Figure 5B). The avian α2, 3-linked SA receptor of ZJ49 adopted a trans conformation, and the glycan rings interacted with the 220-loop of HA (Figure 5D). HAs had a total of five potential glycosylation sites on each monomer (NSTT, NVTV, NKTF, NGTY, NGSM), at HA1 11 (NSTT), 23 (NVTV), 290 (NKTF), HA2 154 (NGTY), and 213 (NGSM). In the structures, four glycans on the stem domain were found, which were 11, 23, 290 sites on HA1 and 154 sites on HA2 (Figure 5C).

## Discussion

The H6-subtype AIVs are widespread in poultry, with a host range extending to mammals such as swine and even human. In recent years, 11 duck samples were infected with H6N6-subtype AIVs in 2017 in Vietnam (Tran et al., 2020). Cui et al. reported that 83 H6 AIVs were isolated from poultry farms between 2014 and 2018 in China, including 38 strains from ducks, 13 from farm environment, and two from geese. These H6 viruses have undergone frequent reassortment, resulting in the formation of 19 different genotypes (Cui et al., 2021). In September 2019, an H6N6-AIV (KNU2019-48) was isolated in South Korea, which was the first reported AIV in Korea. In fact, the H6-subtype viruses had co-circulated in China, Vietnam, and Korea for half a decade (Durairaj et al., 2022). Therefore, the monitoring and prevention of the H6-subtype AIVs, especially H6N6 subtype, should not be taken lightly.

According to the phylogenetic analysis of the HA gene, all H6-subtype AIVs were classified into the gene pools of the Americas and Eurasia. The Eurasian lineage was further divided into three major groups (I, II, and III), ST339-like, ST2853-like, HN573-like (Huang et al., 2010). Our results show that the H6-subtype AIVs were mainly prevalent in the Eurasian continent, and mainly concentrated in China. The ducks were the main host of the H6-subtype AIVs. The evolutionary tree of the 8 segments can be divided into two major branches, namely the Eurasian branch and the North American branch. ST339-like, W312-like, ST2853-like, HN573-like branches are located in G9, G11, G12, and G13 groups. The W312-like branch was closer to the ST2853-like branch, the HN573-like and Taiwan branch was in the same group, and they all evolved from the ST339-like branch and the above results were consistent with previous reports (Zou et al., 2016). In this paper, a new H6N6 strain (ZJ49) was isolated from chickens in Zhejiang Province, China in November 2021. This ZJ49 isolate was located in the G12 group where the ST2853-like branch was also clustered in this group. In recent years, the G12 group became dominant wordwide.

The H6-subtype AIVs spread from North America to Eurasia around 1976. The effective quantity size increased around 1990, reached a peak around 2015, declined after 2015, then kept in a stable level after 2018. However, newly isolated virus strains had been found in China, Vietnam, South Korea and other places. Whether H6-subtype AIV will cause a large-scale re-epidemic in poultry in above mention countries beside North America and Eurasia is unclear. Therefore, it’s important to continuously monitor the genetic characterization and evolution of H6-subtype AIVs to get better control of their epidemic.

The high mutation rate of amino acids enabled AIVs to evolve rapidly, thereby helping them overcome host barriers. Antigenic shift and antigenic drift were two major kinds of antigenic variation occurring in AIVs. Antigenic shift was a result of the reassortment of viral genome segments. To overcome selective pressures and get a better adaptation to the new environments or hosts, the homologous RNA reassortments within segments produced a diverse subtype in most viruses (He et al., 2009; Lam et al., 2013; Ge et al., 2017; Wei and Li, 2018). The H6-subtype AIVs were characterized as LPAIVs, but the reassortment with other influenza virus subtypes may result in the emergence of HPAIVs (Bi et al., 2016; Li et al., 2019). According to the report, A/duck/Guangdong/GD01/2014 was a strain of HPAIV and its NA gene was clustered with some H6N6 viruses circulating in China (Shen et al., 2015). Our results showed that the H6N6-subtype AIVs and H5N6-subtype AIVs recombined frequently. The reassortment analysis predicted that the PB2, PA, and NA genes of ZJ49 were more similar to H5-subtype AIVs. It provided the possibility for H6-subtype AIV recombining with highly pathogenic viruses.

HA protein was a homotrimer, with each monomer containing two polypeptide chains, HA1 and HA2. The receptor-binding site formed a shallow pocket at the tip of the globular domain and comprised three secondary structural elements and one base element (Gamblin and Skehel, 2010). The three secondary elements, namely the 130-loop, the 190-helix, and the 220-loop, formed the edges of the receptor-binding site, and four highly conserved residues (Y98, W153, H183, Y195) formed the base (the numbers correspond to the amino acids in the H3 subtype; Shi et al., 2014). HA proteins exhibited specific binding affinities for the different SA-linked glycoproteins. AIVs preferentially bond to SA-linked terminal oligosaccharides by an α2, 3-linked SA receptor (which is referred to as the avian receptor), whereas human strains favored the α2, 6-linked SA receptor (which is referred to as the human receptor; Shi et al., 2014). Specific amino acid mutations in HA led to a change in receptor-binding preference and thus altered host specificity and tropism of AIVs. The E190V, A222V, G225D, and G228S substitutions of H6-subtype were important to acquire the human receptor-binding capacity (Wang et al., 2015; de Vries et al., 2017; Sun et al., 2017). Our results showed that H6N6-subtype AIVs had a high mutation rate at the A222V site. The ZJ49 strain had no mutations at the E190, G225, and G228 sites, but the A222 site was mutated from A to V leading to a potential risk of its spreading across species. The addition of glycans to the HA was thought to be an important mechanism contributing to antigenic drift (Schulze, 1997; Abe et al., 2004). Oligosaccharides attached to the stem/stalk region of the viral HA tend to be conserved across different viruses strains, whereas those ones attached to the globular head displayed considerable variation in both number and location (Wagner et al., 2000). Glycans in the stalk region were critical for fold and conformation of the HA molecule, while the trimerization, folding, transport of HA to the cell surface, and the sensitivity of HA to changes in pH were impaired by removing these sites from the stalk (Daniels et al., 2003; Tate et al., 2014). According to the three-dimensional structure of this isolate, four conserved glycosylation sites were found in the stem of HA performing less impact on the fold and conception of HA structure.

## Conclusion

This is a study that broadens the knowledge of H6-subtype AIVs from genetic evolutionary, reassortment, and mutations of receptor binding sites. Continuous monitoring and molecular characterization of the H6-subtype AIVs, especially H6N6 subtype, will be required for a better understanding of the evolutionary dynamics of the virus, which can further assist in improving control measures for the diseases.

## Data availability statement

The data presented in the study are deposited in the https://www.ncbi.nlm.nih.gov/nuccore/?term=ON692794:ON692801[accn] repository, accession number ON692794-ON692801.

## Author contributions

MC, YH, and JZ conceived and designed the experiments. MC, YH, and XB performed the experiments. MC and YH analyzed the data. MC and ML wrote the paper. All authors contributed to the article and approved the submitted version.

## Funding

This work was supported by the National Science Foundation of China (grant no. 32192454) and the China Agriculture Research System (grant no. CARS-40-K13).

## Conflict of interest

The authors declare that the research was conducted in the absence of any commercial or financial relationships that could be construed as a potential conflict of interest.

## Publisher’s note

All claims expressed in this article are solely those of the authors and do not necessarily represent those of their affiliated organizations, or those of the publisher, the editors and the reviewers. Any product that may be evaluated in this article, or claim that may be made by its manufacturer, is not guaranteed or endorsed by the publisher.

## Footnotes

^1^ https://services.healthtech.dtu.dk/service.php?NetNGlyc-1.0

^2^ https://swissmodel.expasy.org/interactive/

## Supplementary material

SUPPLEMENTARY TABLE S1 The information of the global distribution of H6NX.

SUPPLEMENTARY TABLE S2 The information of the 8 fragment sequences for construction of the ML tree.

SUPPLEMENTARY TABLE S3 The information of sequences for reassortment analysis of HXN6.

SUPPLEMENTARY TABLE S4 The information of the 8 gene fragments of the maximum similarity reference strain to ZJ49.

SUPPLEMENTARY TABLE S5 The amino acid variation sites of the HA gene of H6N6-subtype AIVs.
