# Supplementary figures and images for: Genetic characterization and evolution of H6N6 subtype avian influenza viruses
Source: Front Microbiol. 2022 Aug 1;13:963218. doi: 10.3389/fmicb.2022.963218 (PMC9376297; doi:10.3389/fmicb.2022.963218)

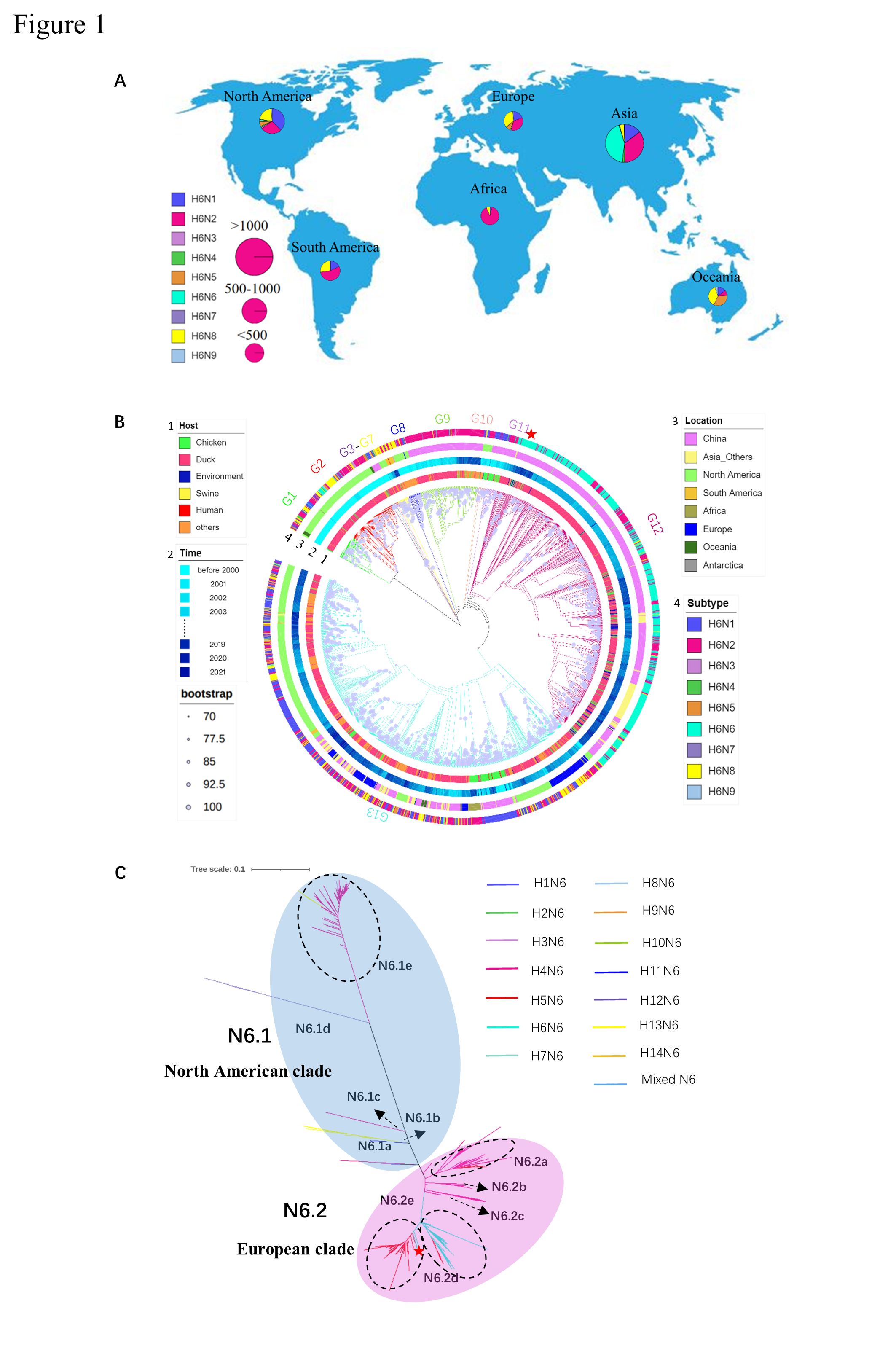

Supplement: Supplementary file 2 [file Data_Sheet_2.ZIP › Figure/Figure1.TIF]

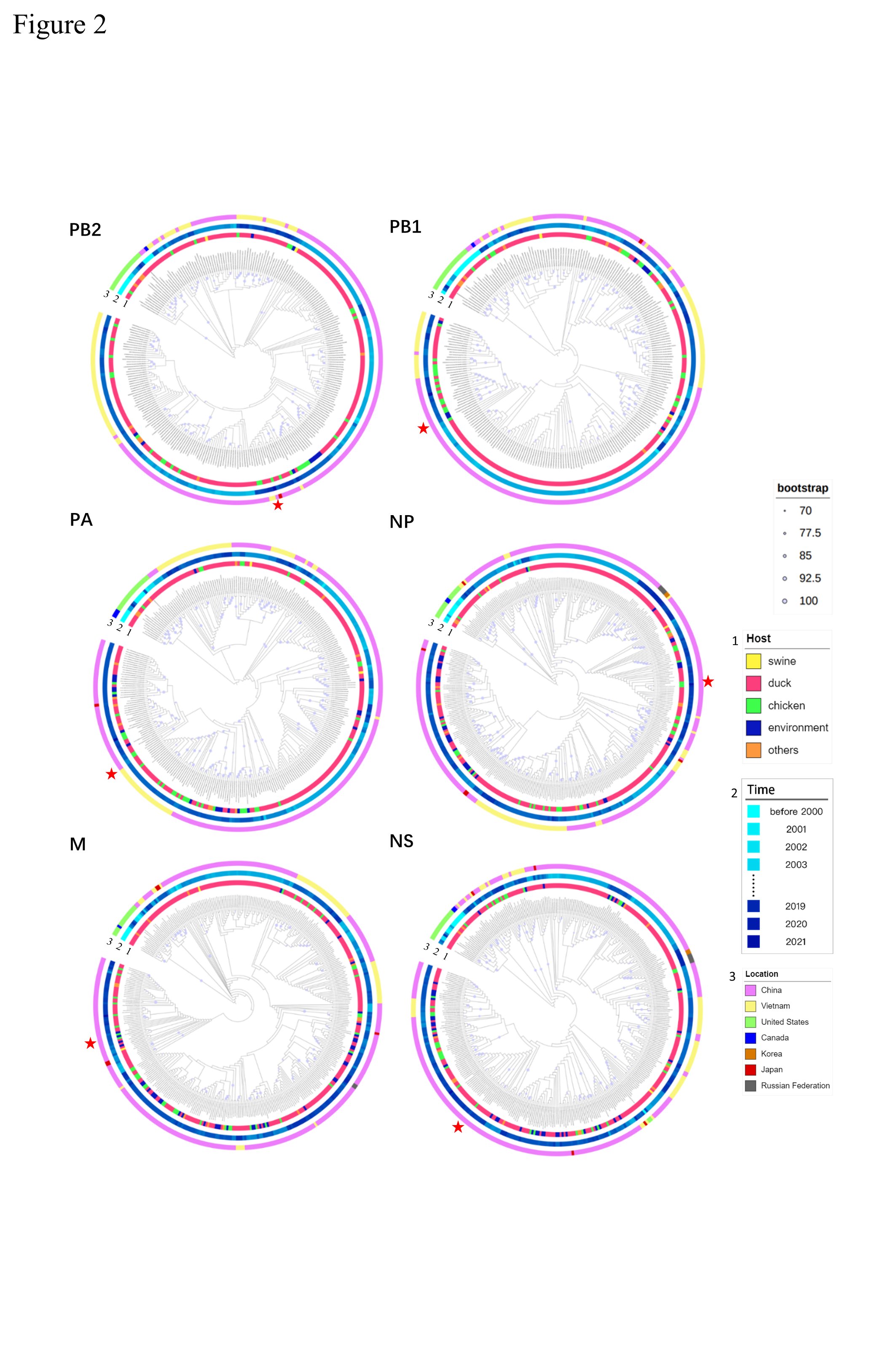

Supplement: Supplementary file 2 [file Data_Sheet_2.ZIP › Figure/Figure2.TIF]

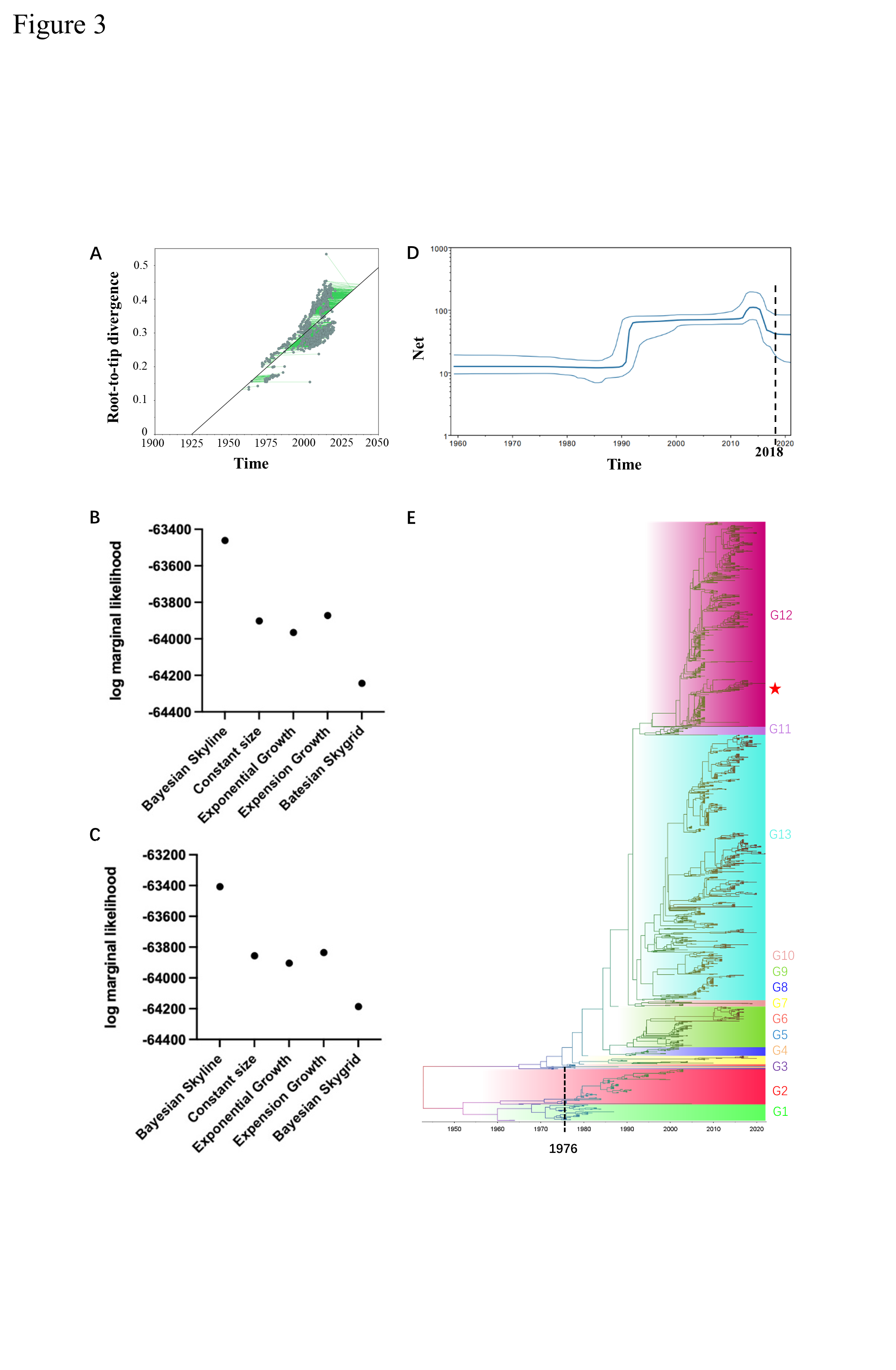

Supplement: Supplementary file 2 [file Data_Sheet_2.ZIP › Figure/Figure3.TIF]

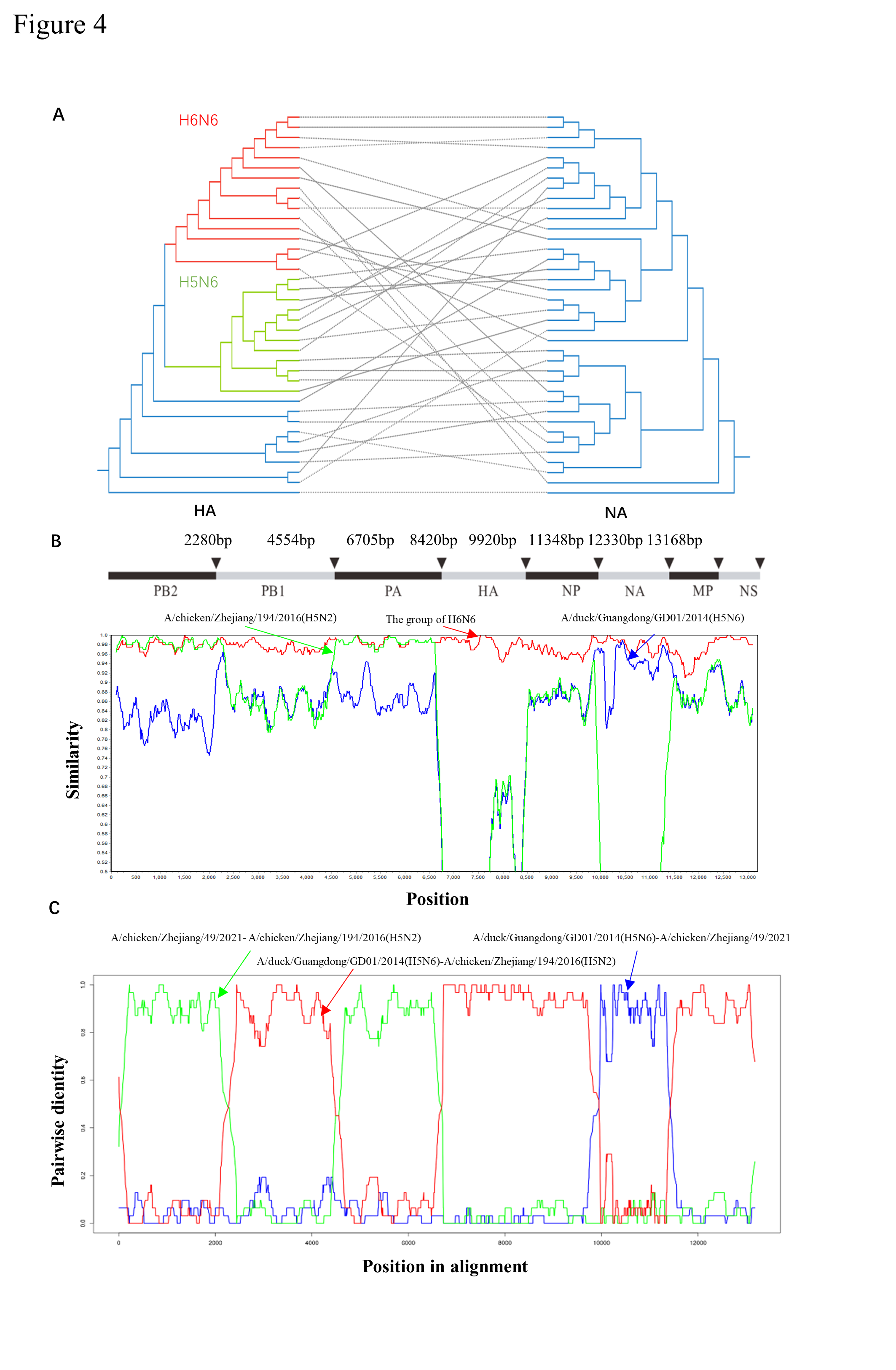

Supplement: Supplementary file 2 [file Data_Sheet_2.ZIP › Figure/Figure4.TIF]

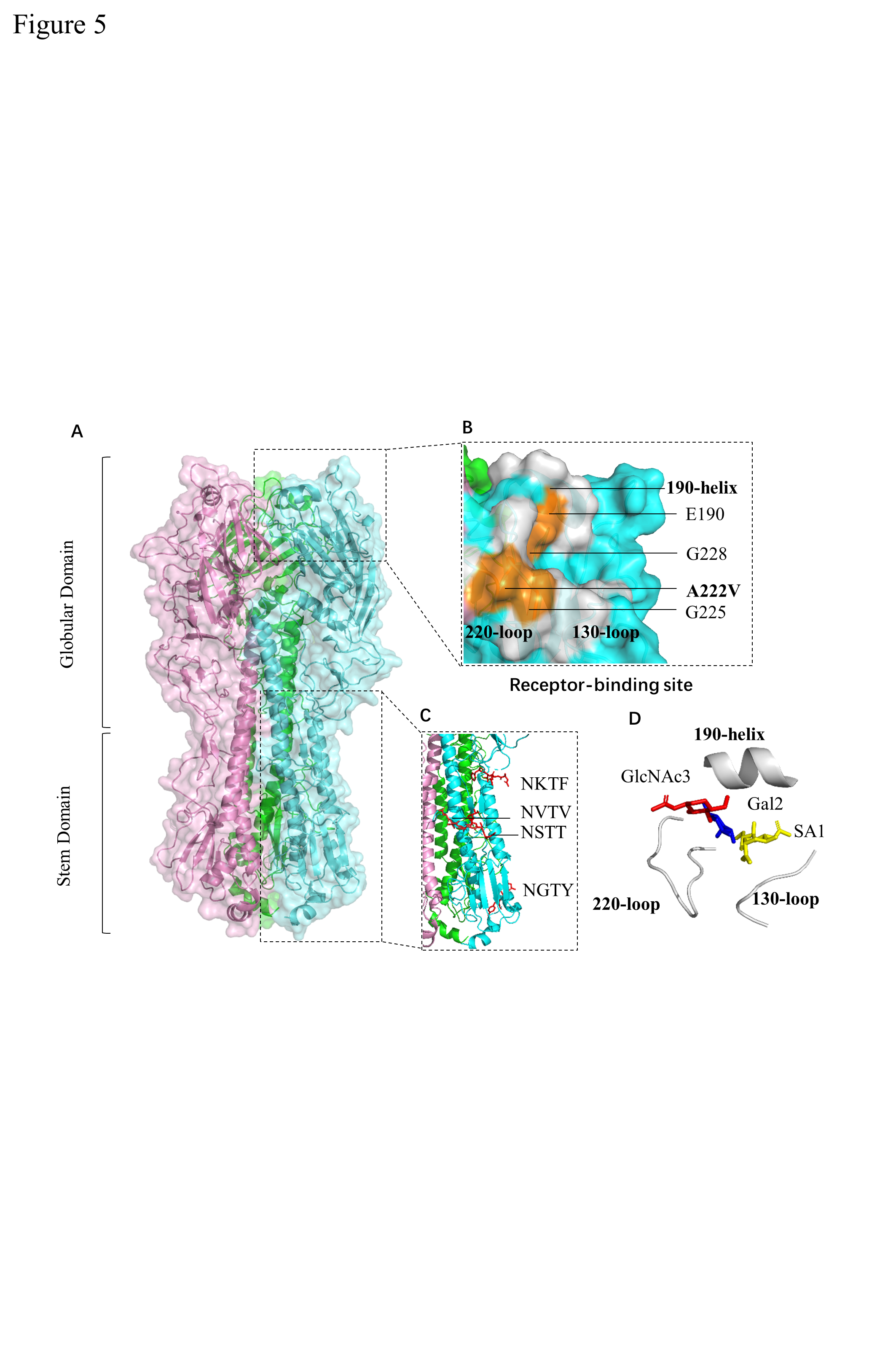

Supplement: Supplementary file 2 [file Data_Sheet_2.ZIP › Figure/Figure5.TIF]
